# Dendrimer-modified gelatin methacrylate hydrogels carrying adipose-derived stromal/stem cells promote cartilage regeneration

**DOI:** 10.1186/s13287-022-02705-6

**Published:** 2022-01-24

**Authors:** Fengyi Liu, Xu Wang, Yuzhou Li, Mingxing Ren, Ping He, Lu Wang, Jie Xu, Sheng Yang, Ping Ji

**Affiliations:** 1 College of Stomatology, Chongqing Medical University, Chongqing, China; 2 Chongqing Key Laboratory of Oral Diseases and Biomedical Sciences, Chongqing, China; 3 Chongqing Municipal Key Laboratory of Oral Biomedical Engineering of Higher Education, Chongqing, China

**Keywords:** Cartilage regeneration, Stem cell therapy, Injectable hydrogel, GelMA, PAMAM

## Abstract

**Background:**

Cartilage defects pose a significant burden on medical treatment, leading to an urgent need to develop regenerative medicine approaches for cartilage repair, such as stem cell therapy. However, the direct injection of stem cells can result in insufficient delivery or inaccurate differentiation. Hence, it is necessary to choose appropriate stem cell delivery scaffolds with high biocompatibility, injectability and chondral differentiation induction ability for cartilage regeneration.

**Methods:**

In this study, the photocrosslinked gelatin methacrylate (GelMA) hydrogel with high cell affinity and plasticity was selected and strengthened by incorporating methacrylic anhydride-modified poly(amidoamine) (PAMAM-MA) to fabricate an adipose-derived stromal/stem cells (ASCs) delivery scaffold for cartilage repair. The physiochemical properties of the GelMA/PAMAM-MA hydrogel, including the internal structure, stability and mechanical properties, were tested. Then, ASCs were encapsulated into the hydrogels to determine the in vitro and in vivo chondrogenic differentiation induction abilities of the GelMA/PAMAM-MA hydrogel.

**Results:**

Compared with the GelMA hydrogel, the GelMA/PAMAM-MA hydrogel exhibited more uniform structure, stability and mechanical properties. Moreover, on the basis of good biocompatibility, the hybrid hydrogel was proven to exert a sufficient ability to promote cartilage regeneration by in vitro three-dimensional (3D) culture of rASCs and in vivo articular cartilage defect repair.

**Conclusions:**

The injectable photocrosslinked GelMA/PAMAM-MA hydrogel was proven to be a capable stem cell carrier for cartilage repair and provides new insight into the design strategy of stem cell delivery scaffolds.

**Supplementary Information:**

The online version contains supplementary material available at 10.1186/s13287-022-02705-6.

## Background

The repair of cartilage defects caused by degenerative disease, congenital anomalies or trauma is a demanding undertaking in the clinic, as cartilage tissue lacks sufficient self-healing capacity [1, 2]. The inherent physiological characteristics of cartilage tissue, including its avascular nature and high matrix-to-cell ratio, lead to the difficulties in developing a successful therapeutic schedule for cartilage regeneration [3, 4]. Conventional surgical procedures for treating cartilage defects, including microfractures and autologous and allogeneic implantation, have certain limitations so that the shape and function of cartilage defects cannot be completely restored [5, 6]. To overcome the difficulties in treatment, stem cell therapy has been developed to provide promising alternative strategies for regenerating cartilage [7, 8].

Among the various kinds of stem cells, adipose-derived stromal/stem cells (ASCs) are considered highly promising in regenerative medicine because of their abundance, ready accessibility with minimally invasive procedures and anti-inflammatory properties [9–11]. However, the direct injection of stem cells has some shortcomings, including excessive cell dosage, induced cell damage or uncontrolled stem cell differentiation [12–14]. Therefore, the delivery of ASCs into the targeted cartilage defect area needs to be carried out in a scaffold-guided manner. In addition, the direction of ASCs differentiation could be controlled by the composition, architecture, physicochemical and mechanical properties of cell delivery vehicles [9, 15, 16]. Accordingly, it is important to select cell delivery scaffolds with appropriate properties for the chondrogenic differentiation induction of ASCs.

As tissue engineering scaffolds, hydrogels have received substantial amounts of attention due to their good biocompatibility, tunable physicochemical properties and structure similar to the three-dimensional (3D) crosslinked hydrophilic network of biological tissues**.**[17, 18]. With these bionic properties, hydrogels are better able to benefit the localization, attachment, proliferation, and desired differentiation of stem cells [19]. To achieve adequate repair of cartilage defects, hydrogels require injectability and in situ gelation, such as the widely used synthetic strategy of photocrosslinking [20–22]. GelMA hydrogels are among the most commonly used photocrosslinked hydrogels with high biocompatibility to mimic the native extracellular environment [23, 24]. However, the potential applications of GelMA hydrogels are hindered due to their low mechanical properties and excessive degradation speed, which prevents the material from utility in the regeneration of load-bearing tissues such as cartilage [25, 26]. Therefore, it is essential to combine GelMA with other materials to ameliorate these limitations.

Poly(amidoamine) (PAMAM) dendrimers are spherical hyperbranched polymers with specific controllable sizes and easily modified surfaces [27, 28]. Owing to these outstanding properties, PAMAM dendrimers have long been used in the fields of biomedicine such as drug delivery, molecular imaging and gene therapy [29–31]. Moreover, PAMAM dendrimer incorporated materials have shown promising results when investigated in skin and corneal tissue engineering applications [32, 33]. In addition, we have previously introduced PAMAM dendrimer into the hybrid poly(lactic acid)-b-poly(ethylene glycol)-b-poly(lactic acid) (PEG-LA-DA) hydrogel system for bone tissue engineering [34]. Hence, PAMAM has great potential for the building composite tissue engineering scaffolds for cartilage regeneration.

**Fig. 1:**
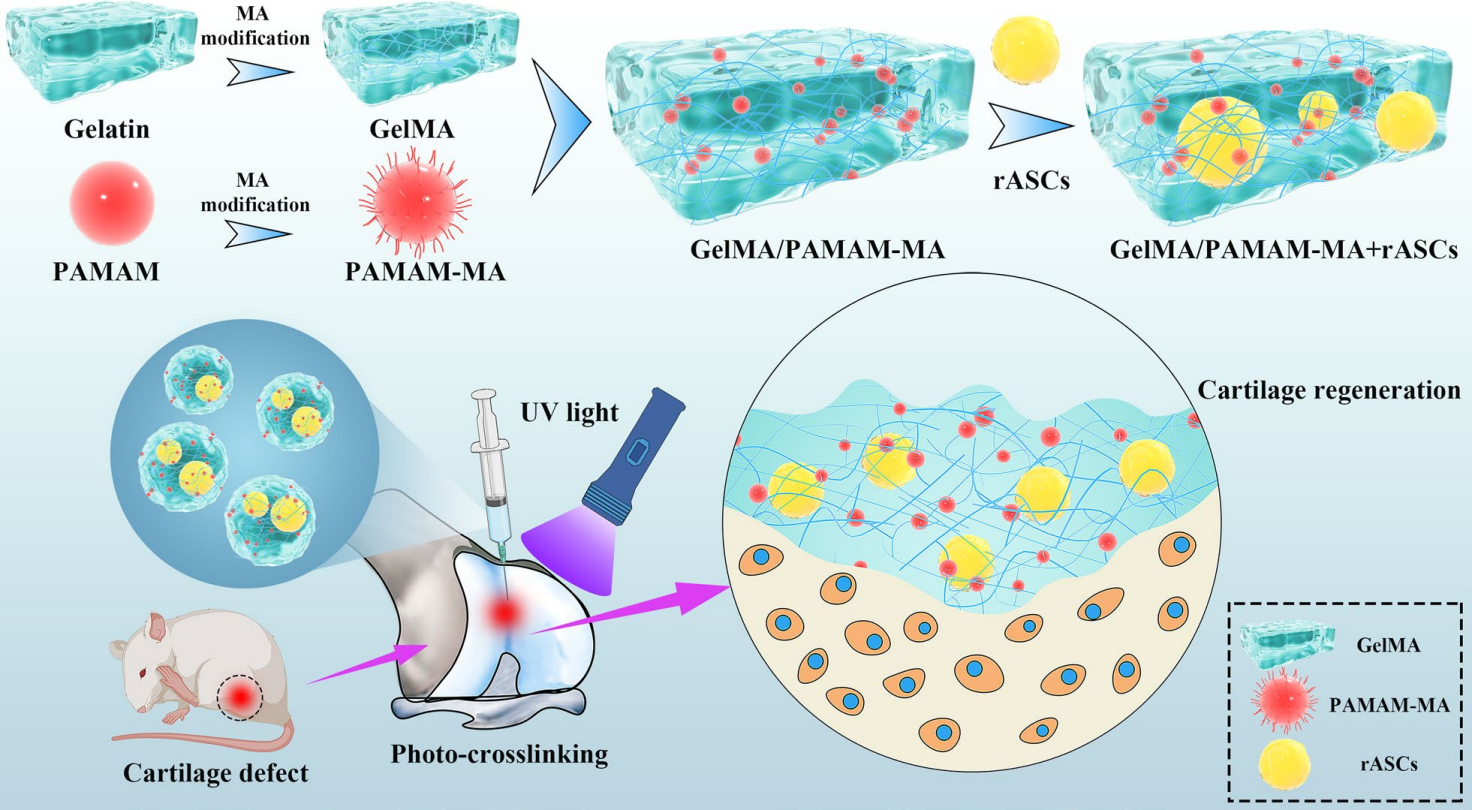
Schematic diagram of the injectable stem cell-laden photocrosslinked GelMA/PAMAM-MA hydrogel for cartilage regeneration

In this research, GelMA and methacrylic anhydride (MA)-modified PAMAM (PAMAM-MA) were used to fabricate an injectable stem cell-laden photocrosslinked hydrogel for cartilage regeneration (Fig. 1). GelMA hydrogels constituted the control group. To analyze the effect of the composition of hydrogels on their physiochemical properties, we compared the structure, stability and mechanical strength of two groups of GelMA/PAMAM-MA hydrogels through various tests. We then performed a series of in vitro and in vivo studies to verify whether the addition of PAMAM-MA can induce the chondrogenic differentiation of stem cells and repair cartilage defects properly. ASCs were encapsulated in two groups of hydrogels for in vitro stem cell chondrogenic differentiation analysis by detecting the expression of chondrogenic-related genes and proteins. For in vivo cartilage defects, we constructed a knee articular cartilage defect model for further therapy. Two groups of injectable stem cell-laden hydrogels were used for in situ treatment to analyze their cartilage defect repair abilities.

## Methods

### Synthesis of GelMA and PAMAM-MA

The synthesis of GelMA and PAMAM-MA followed protocols described previously [26, 35]. Gelatin powder from cold-water fish skin (Sigma–Aldrich, USA) was dissolved in phosphate-buffered saline (PBS; HyClone, USA) at a concentration of 100 mg/mL at 50 °C. Methacrylic anhydride (MA, Aladdin, China) was added to the gelatin solution at a rate of 0.5 mL/min (20%, w/v). The mixture was reacted for 3 h under stirring conditions at 50 °C. The reaction was then terminated by the addition of excessively warm PBS. The GelMA solution was dialyzed against deionized water for 7 days at 40 °C to remove excess residual small molecules, and then the GelMA solution was filtered, freeze-dried and stored at − 20 °C. PAMAM (Weihai CY Dendrimer Technology Co., Ltd., China) was modified with MA in accordance with the same protocol above to synthesize PAMAM-MA.

### Fourier transform infrared (FTIR) spectroscopy analysis

FTIR spectroscopy was performed to verify whether GelMA and PAMAM-MA were synthesized successfully. Gelatin, PAMAM, GelMA and PAMAM-MA were tested to compare the composition of two targeted polymers before and after their modification with MA. To obtain the FTIR spectra, FTIR analysis was performed via an FITR spectrometer (Nicolet iS50, Thermo Fisher Scientific, USA) by recording 256 scans, each with a spectral width ranging from 500 to 4000/cm at a spectral resolution of 4/cm.

### Formation of GelMA/PAMAM-MA hydrogels

Lyophilized GelMA and PAMAM-MA were dissolved in photoinitiator solution separately at a concentration of 100 mg/mL. To prepare the photoinitiator solution, 2-hydroxy-1(4-(hydroxyethoxy)pheny)-2-methyl-1-propanone (Irgacure 2959, Sigma, USA) was dissolved in PBS at a concentration of 2 mg/mL under ultrasonic vibration at 50 °C. GelMA and PAMAM-MA solutions were then mixed to prepare the prepolymer solutions. The final concentration of GelMA in prepolymer solutions was 50 mg/mL, and PAMAM-MA solutions were prepared at the final concentrations of 0 or 20 mg/mL. The prepolymer solutions were exposed to 365 nm ultraviolet (UV) light for 30 s to synthesize hydrogels, after which they were soaked in PBS for further use.

### Hydrogel pore sizes and internal structure test

The effect of the composition of hydrogels on the pore size and network structure of hydrogels was characterized by scanning electron microscopy (SEM) and frozen sectioning. GelMA/PAMAM-MA hydrogels were frozen at − 80 °C and then lyophilized before being sputter coated with Au–Pd (2 nm) and examined via SEM (Hitachi-SU8010, Japan). For frozen section detection, two groups of hydrogels were immersed in PBS for 48 h, embedded in optimal cutting temperature compound (OCT, Sakura Finetek, USA), and then frozen at − 80 °C overnight. The hydrogels were sliced into 10 μm-thick sections by using a cryostat (Leica, Germany) and then stained with hematoxylin (Solarbio, China). The pore sizes of the hydrogels were measured with ImageJ software (NIH, USA).

### Swelling ratio analysis

The swelling ratio of GelMA/PAMAM-MA hydrogels was determined by measuring the weight change in the hydrogel before and after dehydration to analyze the effect of hydrogel components on their stabilities. Hydrogels were incubated in PBS at room temperature for 48 h, and the initial weight (Wi) was weighed. The hydrogels were then freeze dried for 24 h, and the dry weight (Wd) was measured. The following equation was used to calculate the swelling ratio: swelling ratio = (Wi − Wd)/Wd × 100%.

### In vitro degradation tests

The degradation of GelMA and GelMA/PAMAM-MA hydrogels was performed in vitro by immersing hydrogels into PBS solution containing 1 unit/mL collagenase type II (Solarbio, China). The initial weight of the lyophilized hydrogels (n = 4) was measured and recorded, and hydrogels were immersed in 1 mL of degradation solution and incubated under shaking at 37 °C. The degraded hydrogels were washed twice and lyophilized at each set time point (after 12 h, 1 day, 2 days, 4 days and 7 days). The weight of the freeze-dried hydrogels was measured. The degradation solution was replaced by freshly prepared degradation solution.

### Mechanical test

The compressive strength of two groups of GelMA/PAMAM-MA hydrogels was measured to analyze the effect of hydrogel composition on the mechanical properties of hydrogels. The compressive modulus of GelMA and GelMA/PAMAM-MA hydrogel disks (thickness = 2 mm, diameter = 5 mm) was determined by using a universal testing machine (MSD, USA). The compressive stress−strain curves were obtained at a strain rate of 0.2 mm/min. The compressive modulus was determined by taking the slope of the linear region of the stress–strain curves at 0–20% strain.

### Isolation and identification of rat adipose-derived stromal/stem cells

Rat adipose-derived stromal/stem cells (rASCs) were isolated and identified as described previously [36]. Briefly, rat adipose tissues of the groin were isolated and washed three times with PBS containing 1% penicillin–streptomycin (HyClone, USA). Adipose tissues were then cut and digested with type I collagenase (Sigma, USA), after which they were dissolved in Dulbecco’s modified Eagle’s medium (DMEM; Gibco, USA) for 1 h at 37 °C. After centrifugation at 1000 rpm for 5 min, the cell pellet was suspended DMEM that included 10% fetal bovine serum (HyClone, Australia) and 1% penicillin–streptomycin. The cell suspension was filtered through a 70 μm cell filter and centrifuged for another 5 min. Cell pellets were suspended in growth medium and incubated in a CO_2_ incubator at 37 °C.

Third generation rASCs were used for identification. Flow cytometry (FCM) was used to detect the expression of surface antibodies related to stem cell identification, including CD90, CD44, CD45 and CD31 (BioLegend, USA). Cells were digested and centrifuged at 1000 rpm and washed with PBS. After incubation with antibodies (1:100) at 4 °C for 30 min, the cells were subjected to FCM (Becton Dickinson, USA). The results were analyzed with FlowJo software (FlowJo, USA). rASCs were also identified by osteogenic, adipogenic and chondrogenic differentiation. Third-generation rASCs were induced in osteogenic differentiation medium (Cyagen, China) for 7 days and stained with an alkaline phosphatase staining kit (Beyotime Biotechnology, China) to verify the osteogenic differentiation of stem cells. rASCs were induced in adipogenic differentiation medium (Cyagen, China) for 21 days and stained with an oil red O staining kit (Solarbio, China) to verify adipogenesis. For chondrogenic differentiation, rASCs were induced in chondrogenic differentiation medium (Cyagen, China) for 14 days. Cell pellets were embedded in OCT for frozen sectioning. After storage in a − 80 °C refrigerator overnight, cell pellets were cut into 10 μm sections and stained with Alcian Blue solution. The staining results were observed and imaged by inverted microscopy (Olympus, Japan).

### In vitro 3D cell culture

For 3D cell culture, rASCs were resuspended in two groups of GelMA/PAMAM-MA prepolymer solutions at a density of 1 × 10^7^ cells/mL. The mixture was added to polydimethylsiloxane (PDMS) molds and exposed to 365 nm UV light for 30 s. The synthesized hydrogels were immersed in growth medium and cultured in a CO_2_ incubator at 37 °C.

### Live/dead cell staining

To assess the cell viability of rASCs encapsulated in GelMA/PAMAM-MA hydrogels, live/dead cell staining was performed. After rASCs were encapsulated in two groups of GelMA/PAMAM-MA hydrogels for 1 day, 4 days and 7 days, the hydrogels were washed with PBS twice and stained with calcein-AM/PI dyeing solution (Bestbio, China) for 20 min. Subsequently, the hydrogels were washed with PBS three times and observed by confocal microscopy (CLSM, Zeiss LSM 9100, Germany). ImageJ software was used to count and analyze the numbers of living cells and dead cells.

### Cytoskeleton/nucleus fluorescent staining

To evaluate the cell spreading status, rASCs in GelMA/PAMAM-MA hydrogels were measured with cytoskeleton/nucleus fluorescent staining after 1, 4 and 7 days of cell culture. Alexa Fluor 488-conjugated phalloidin (Invitrogen, USA) and 4′,6-diamidino-2-phenylindole (DAPI; Invitrogen, USA) were used for cytoskeletal and nuclear staining, respectively. The samples were fixed in 4% paraformaldehyde (PFA; Sigma, USA) for 10 min and washed with PBS three times. The hydrogels were incubated in phalloidin solution (1:300 dilution) for 2 h at room temperature. For DAPI/cell nuclei staining, DAPI solution (1:1000 dilution) was used to incubate hydrogels for 30 min. The hydrogels were then measured by confocal microscopy to observe the extent of cell spreading.

### Quantitative real-time polymerase chain reaction (qPCR) analysis

qPCR was performed to quantitatively evaluate the messenger ribonucleic acid (mRNA) expression level of chondrogenic differentiation genes in rASCs encapsulated in two groups of hydrogels, including sex determining region Y-box transcription factor 9 (SOX9), aggrecan (ACAN), type II collagen (Col-II) and type X collagen (Col-X). rASCs were encapsulated in hydrogels and cultured in chondrogenic induction medium (Cyagen, China) for 7 days. Total RNA was extracted and examined by a NanoDrop spectrophotometer (Thermo Fisher Scientific, USA) to determine the concentration. A PrimeScript RT Master Mix Kit (Takara, Japan) was used to reverse‑transcribe RNA to complementary DNA (cDNA) according to the manufacturer’s instructions. The cDNA of target genes was mixed with SYBR Green PCR Master Mix (Takara, Japan) and loaded into a Real-Time PCR System (Bio–Rad, USA) to analyze the expression levels of chondrogenesis-related genes; glyceraldehyde-3-phosphate dehydrogenase (GAPDH) was used as the housekeeping gene. The sense and antisense primer sequences of the above genes are listed in Additional file 1: Supplementary information, Table S1. The relative gene expression levels were analyzed using the comparative Ct (ΔΔCt) method.

### Safranin-O staining

To analyze the effect of hydrogel components on the deposition of cartilage-specific extracellular matrix (ECM) in hydrogels, safranin-O staining was used for detection. After culturing in chondrogenic induction medium for 14 days, two groups of GelMA/PAMAM-MA hydrogels were rinsed with PBS, fixed in 4% PFA for 10 min and washed with PBS three times. Hydrogels were then embedded within OCT and stored in a − 80 °C refrigerator overnight prior to frozen sectioning. The hydrogels were sectioned into 10 μm-thick sections and stained with safranin-O dyeing solution (Solarbio, China) by following the manufacturer’s instructions. The staining results were recorded via inverted microscopy.

### Immunofluorescence (IF) staining

IF staining was used to study the expression of the chondrogenic-associated protein SOX9 in rASCs. After culturing in chondrogenic induction medium for 21 days, cell-laden hydrogels were fixed in 4% PFA for 10 min and washed with PBS three times. Hydrogels were blocked with 5% donkey serum and incubated with primary antibodies (SOX9, Abcam, USA, ab185966, 1:300) overnight at 4 °C. Afterward, the hydrogels were incubated with secondary antibodies (Alexa Fluor 488-AffiniPure Donkey anti-rabbit IgG, Invitrogen, USA, #a21206, 1:300) for 1 h at room temperature. Finally, the hydrogels were stained with DAPI solution and measured via confocal microscopy.

### In vivo rat cartilage defect repair study

In this study, 6-week-old female Sprague Dawley rats (200–250 g weight, Chongqing, China) were used and treated with standard surgical procedures, which were conducted in accordance with protocols approved by the Ethics Committee of the Stomatological Hospital of Chongqing Medical University. Rats were anesthetized with isoflurane and defects (1.5 mm in diameter, 1.0 mm in depth) were created by a dental drill on the surface of rat knee joints. Hydrogel prepolymer mixture solutions loaded with rASCs were injected into defects and exposed to 365 nm UV light for 30 s. Four groups were established a normal control group (untreated group), blank control group (defects unrepaired group), GelMA + rASCs group and GelMA/PAMAM-MA + rASCs group.

### In situ observation of implanted rASCs

To trace the rASCs implanted into the cartilage defects, we used green fluorescent protein (GFP)-labeled adenovirus (GeneChem, China) to transfect the cells. The transfected rASCs were encapsulated into GelMA/PAMAM-MA hydrogel and injected into the defect areas. At 1 week, 2 weeks and 4 weeks after implantation, the rats were sacrificed and the cartilage defect areas were median sectioned and stained with DAPI solution to observe the rASCs in the defect areas.

### Histological analysis of repaired cartilage defect tissues

At 8 weeks after repair, the rats were sacrificed. Rat knee joints were separated, washed with PBS and imaged. Samples were then fixed in 4% PFA solution for 24 h at 4 °C. All samples were decalcified by 20% ethylenediaminetetraacetic acid (EDTA) solution for 2 months, dehydrated, embedded in paraffin, and sectioned into 5 μm-thick sections. To evaluate the extent of tissue reconstruction, sections of all groups were stained with hematoxylin and eosin (H&E) staining solutions (Solarbio, China) and a safranin-O/fast green staining kit (Solarbio, China). Histological points of the repaired cartilage defect tissues were scored by 3 independent blinded observers using the International Cartilage Repair Society (ICRS) visual histological assessment [37].

### Immunohistochemical (IHC) staining

For IHC staining, sections were deparaffinized and digested with pepsin for 20 min at 37 °C for antigen retrieval. The sections were then incubated in 3% hydrogen peroxide solution for 10 min to eliminate endogenous peroxidase activity at room temperature. The sections were blocked with 5% goat serum and incubated with proper dilutions of primary antibodies overnight at 4 °C. The following primary antibodies were used: SOX9 (Abcam, USA, ab185966, 1:300) and Col-II (Santa Cruz, USA, sc-52658, 1:300). Afterward, the sections were incubated with secondary antibodies (Bioss antibodies, China: goat anti-rabbit IgG H&L antibody, #bs-0295G, 1:300; goat anti-mouse IgG H&L antibody, #bs-0296G, 1:300) for 60 min at room temperature. The sections were then stained with the chromogenic agent 3,3′-diaminobenzidine (DAB) to visualize the specific expression of targeted proteins. Finally, the sections were stained with hematoxylin for cell nuclear localization.

### Statistical analysis

All experiments were repeated three times to verify the results. Data analysis was performed via GraphPad Prism 7.0 (GraphPad Software, USA). Statistical analysis was evaluated using Student’s t test for two-group comparisons and using one-way ANOVA with Tukey’s post hoc test for multigroup comparisons. A value of *p* < 0.05 was considered statistically significant. The results were presented as the mean ± Standard Deviation (SD).

## Results

### FTIR characterization

**Fig. 2:**
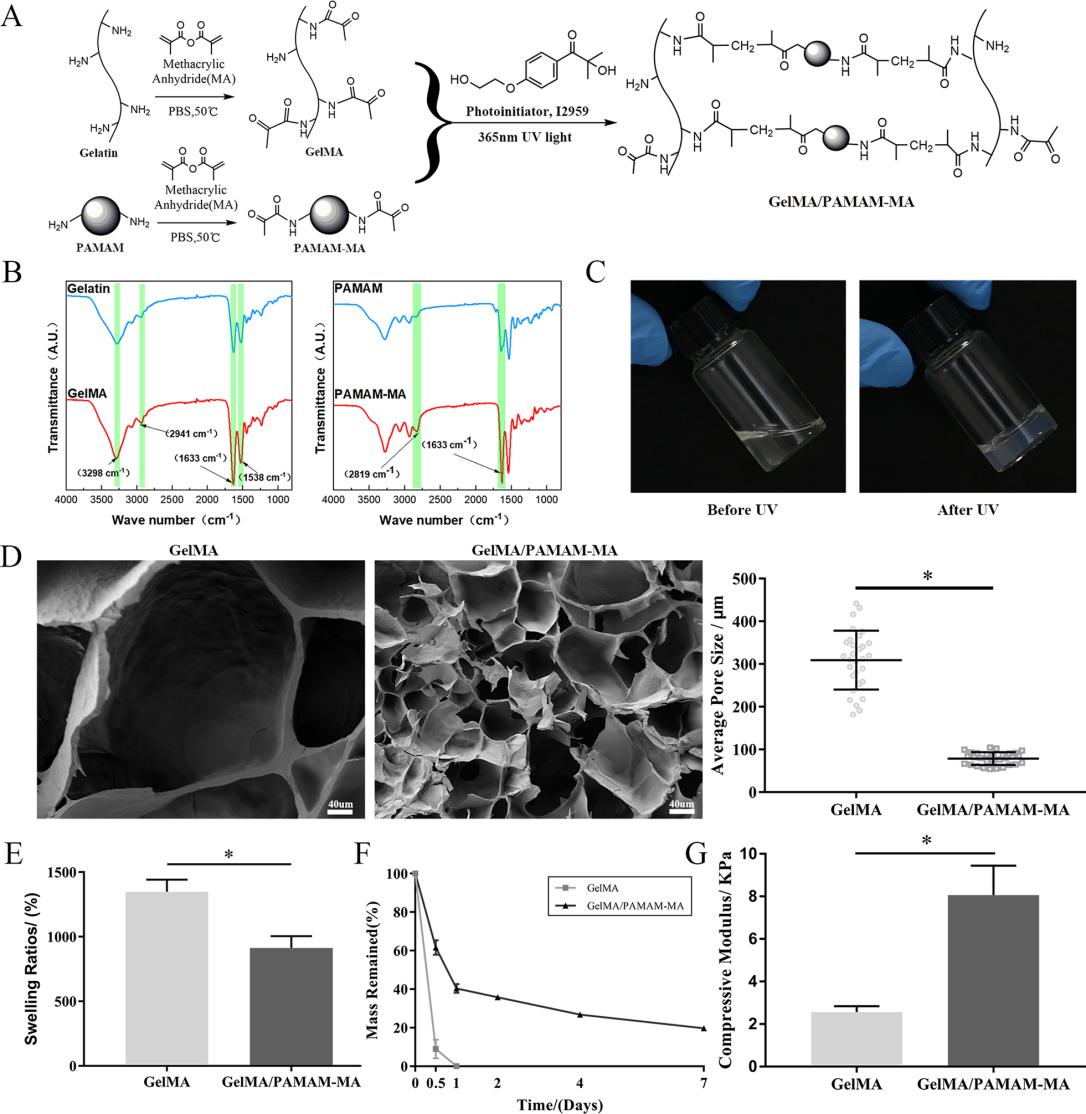
Physiochemical property analysis of GelMA/PAMAM-MA hydrogels. **A** Schematic illustration of the preparation of photocrosslinked GelMA/PAMAM-MA hydrogel. **B** FTIR spectroscopy of gelatin, GelMA, PAMAM and PAMAM-MA. **C** Optical images of the GelMA/PAMAM-MA hydrogel before and after 365 nm UV light irradiation. **D** SEM characterization pictures and statistical analysis of GelMA and GelMA/PAMAM-MA hydrogel pore sizes. Scare bars: 40 μm. **E**. Swelling ratio test of two groups of hydrogels. **F** In vitro degradation test of GelMA and GelMA/PAMAM-MA hydrogel. **G** Compressive modulus analysis of GelMA and GelMA/PAMAM-MA hydrogels. Error bar: Mean ± SD, * represents* p* < 0.05

The synthesis of GelMA and PAMAM-MA is described in Fig. 2A. Gelatin and PAMAM were modified by MA at 50 °C. FTIR was performed to verify the MA modification of gelatin and PAMAM (Fig. 2B). The intensities of the typical amide bonds at 1633/cm and 1538/cm in the spectra of gelatin and GelMA varied. Moreover, the stretching vibration peak of the N–H group was observed at 3297/cm, and the peak at 2941/cm was a C–H stretching peak. For the spectra of PAMAM and PAMAM-MA, a change in the peak at 1633/cm was observed for the typical amide bands and the peak at 2819/cm was attributed to C–H stretching. Taken together, these changes were caused by the modification of amino groups by MA to form new amide bonds, and were evidence of successful modification of gelatin and PAMAM with MA.

### Synthesis and internal structure measurement of GelMA/PAMAM-MA hydrogels

As shown in Fig. 2A and C, the GelMA/PAMAM-MA hydrogel was synthesized after exposure to 365 nm UV light for 30 s. We examined the pore sizes and internal structures of two groups of the two hydrogels under two different dry and wet conditions through SEM (Fig. 2D) and frozen section inspections (Additional file 1: Supplementary information, Fig. S1). The internal cross-linked network of the GelMA hydrogel was irregular and dispersed in both the freeze-dried state and the wet state. Correspondingly, the GelMA/PAMAM-MA hydrogel exhibited a well-distributed and integrated internal network. The pore sizes of the two groups of hydrogels were analyzed and compared, while GelMA/PAMAM-MA hydrogels presented smaller average pore sizes and more compact and uniform structures.

### Stability of GelMA/PAMAM-MA hydrogels

The swelling ratios were tested to evaluate the stability of the injectable photocrosslinked hydrogels. By measuring the wet and dry weights of hydrogels, we calculated and analyzed the swelling ratios. The results in Fig. 2E demonstrate that the swelling ratio of the hydrogels declined significantly with the addition of PAMAM-MA, which could be attributed to changes in the pore sizes of the hydrogels. Furthermore, the results of in vitro degradation tests of the two groups of hydrogels showed that the GelMA hydrogel completely degraded within 24 h, while the GelMA/ PAMAM-MA hydrogel could retain approximately 20% of the initial mass when it degraded for 7 days (Fig. 2F). These results indicated that PAMAM-MA can effectively improve the stability of hydrogels.

### Mechanical properties of GelMA/PAMAM-MA hydrogels

Mechanical properties of hydrogels profoundly regulate the differentiation of stem cells. Therefore, we introduced PAMAM-MA into the GelMA hydrogel, aiming at adjusting the mechanical properties of hydrogels to regulate the differentiation of rASCs. The mechanical properties of the two groups of GelMA/PAMAM-MA hydrogels were determined by measuring the compression modulus. We tested the compressive strength of hydrogels by using a universal testing machine. As shown in Fig. 2G, statistical analysis revealed that the compression modulus of the GelMA/PAMAM-MA hydrogel (8.06 ± 1.39 kPa) increased significantly with the addition of PAMAM-MA compared with that of the GelMA hydrogel (2.56 ± 0.26 kPa).

### Identification of rASCs

**Fig. 3:**
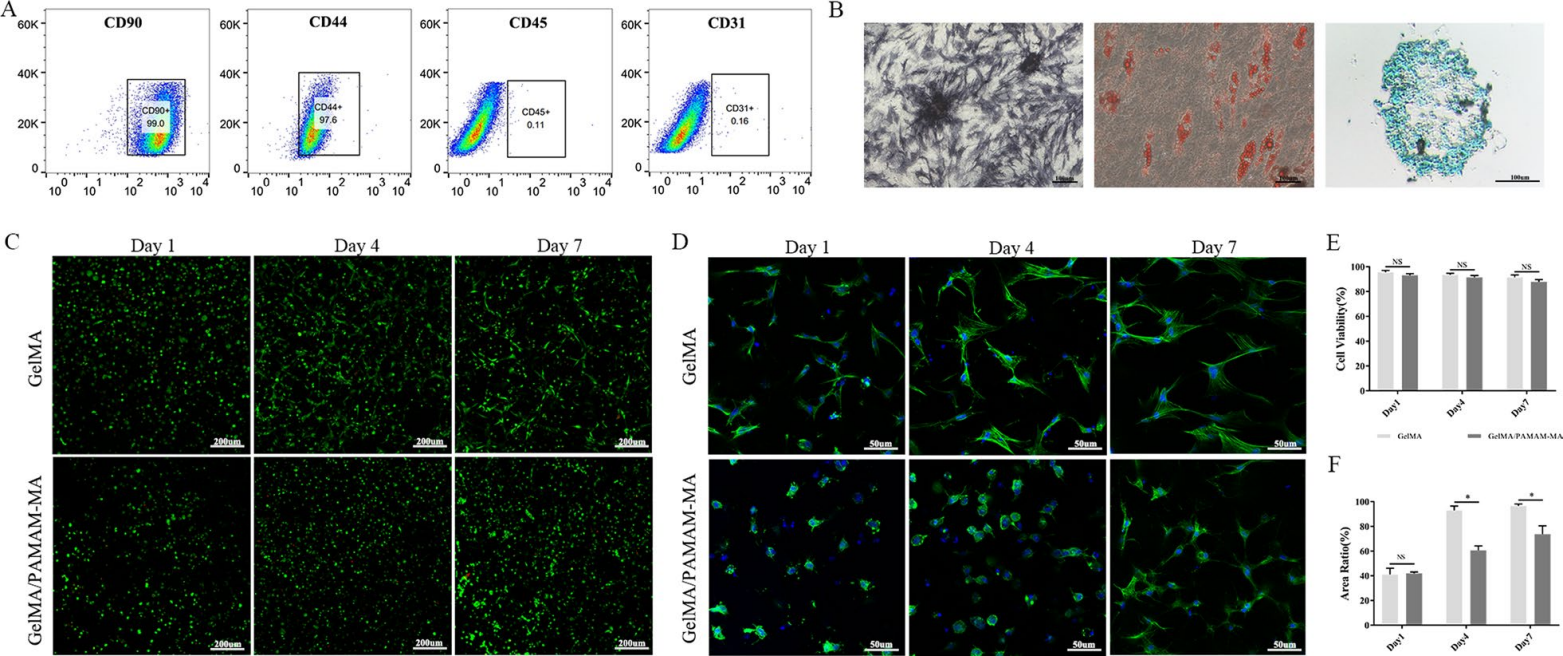
Identification of rASCs and in vitro biocompatibility evaluations. **A** FCM analysis of the expression of stem cell identification-related antibodies in rASCs. **B** Representative images of the multidifferentiation ability test of rASCs. Left, ALP staining, middle, Oil red O staining, right, Alcian blue staining. Scare bars: 100 μm. **C** Representative images of live/dead rASCs cultured in GelMA and GelMA/PAMAM-MA hydrogels on days 1, 4 and 7. Green fluorescence indicates live cells, and red fluorescence indicates dead cells. Scare bars: 200 μm. **D** Representative images of phalloidin/DAPI fluorescence images of rASCs cultured in two groups of GelMA/PAMAM-MA hydrogels after 1 day, 4 days and 7 days. Green fluorescence indicates the cytoskeleton, and blue fluorescence indicates the nucleus. Scare bars: 50 μm. **E** Quantification of the live and dead cells. **F** Quantification of the cell spreading areas of rASCs cultured in two groups of hydrogels. Error bar: Mean ± SD, * represents *p* < 0.05

The FCM analysis results in Fig. 3A revealed that rASCs were positive for the expression of mesenchymal stem cell-related surface antigens, including CD90 (99.0%) and CD44 (97.6%). In addition, rASCs were negative for the expression of the endothelial-related marker CD31 (0.16%) or hematopoietic-related marker CD45 (0.11%). The multidifferentiation ability of rASCs is shown in Fig. 3B. The positive ALP staining results revealed the osteogenic differentiation ability of rASCs after osteogenic induction culture. Oil red O staining also revealed visible accumulation of intracytoplasmic lipid droplets, which proved the adipogenic differentiation ability of rASCs. Moreover, Alcian blue staining of rASCs proved their chondrogenic differentiation ability.

### In vitro biocompatibility tests

To evaluate the biocompatibility of the two groups of hydrogels, we measured the cell viabilities of rASCs encapsulated in GelMA and GelMA/PAMAM-MA hydrogels by staining with calcein-AM/PI dyeing solution and observed them via confocal microscopy. Live/dead cell staining images of ASCs encapsulated in hydrogels after culture for 1, 4 and 7 days displayed a high rate of cell viability and uniform distribution (Fig. 3C). We counted and analyzed the living and dead cell numbers, and the results showed that cell viabilities of rASCs encapsulated in the two groups of hydrogels remained above 80% at all time points, with no significant differences (Fig. 3E).

We examined the spreading of rASCs in GelMA and GelMA/P AMAM-MA hydrogels by cytoskeleton/nuclei fluorescent staining at day 1, day 4 and day 7. rASCs encapsulated in hydrogels gradually spread as the culture time increased (Fig. 3D). When comparing the spread of rASCs between the two groups of hydrogels, we found that the extent of cell spreading decreased in the GelMA/PAMAM-MA hydrogels. The cell spreading areas of rASCs were then quantified using Image-Pro Plus software, and statistically significant decreases were observed in GelMA/PAMAM-MA hydrogels at day 4 and day 7 (Fig. 3F). rASCs in the GelMA/PAMAM-MA hydrogel presented a decreased spreading area and spherical morphology, similar to that of chondrocyte phenotype, which might be beneficial to chondrogenesis of rASCs.

### In vitro chondrogenesis study

**Fig. 4:**
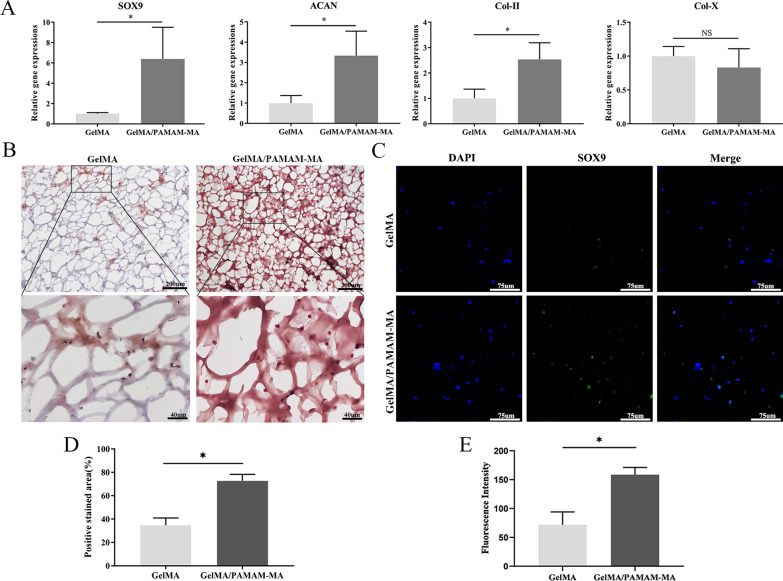
In vitro chondrogenic differentiation analysis of 3D-cultured rASCs. **A** Quantitative real-time PCR analysis of the expression of chondrogenic differentiation-related genes in rASCs encapsulated in GelMA and GelMA/PAMAM-MA hydrogels. **B** Representative images of safranin-O staining after 14 days of chondrogenic induction culture. Scale bars: above, 200 μm; below, 40 μm. **C** Representative images of immunofluorescence staining of SOX9 in rASCs encapsulated in two groups of hydrogels after 21 days of chondrogenic induction culture. Scale bars: 75 μm. **D** Quantification of the positively stained area of GAGs in the two groups of hydrogels. **E** Quantification of SOX9 expression in the two groups of hydrogels. Error bar: Mean ± SD, * represents *p* < 0.05

We first analyzed and compared the ability of two groups of hydrogels to regulate the chondrogenic differentiation of rASCs by detecting the expression of chondrogenic-related genes in rASCs, including the chondrogenic phenotype genes SOX9, Col-II, ACAN and Col-X. The qPCR analysis results showed varied gene expression levels of rASCs encapsulated in GelMA and GelMA/PAMAM-MA hydrogels after 1 week of in vitro differentiation (Fig. 4A). The expression of SOX9, ACAN and Col-II significantly increased in the GelMA/PAMAM-MA hydrogel, while the expression of the hypertrophic cartilage-related gene Col-X showed no significant difference between the two groups.

Furthermore, we carried out frozen sectioning and stained rASCs with safranin-O dye after 2 weeks of chondrogenic induction culture to visualize the spatial distribution of glycosaminoglycans (GAGs) in the two groups of hydrogels. Representative images showed that the deposition of GAGs increased significantly in GelMA/PAMAM-MA hydrogels compared with GelMA hydrogel (Fig. 4B). The positive stained area of GAGs was semiquantitatively analyzed by ImageJ software, and the result showed a significant difference between the two groups of hydrogels (Fig. 4D).

In addition, we performed immunofluorescence staining of SOX9 protein in rASCs encapsulated in two groups of hydrogels after 21 days of chondrogenic induction culture, and more positively stained cells could be observed in GelMA/PAMAM-MA hydrogels (Fig. 4C). The semiquantitative analysis of the immunofluorescence staining images by ImageJ software revealed a significant difference in the expression of SOX9 between the two groups (Fig. 4E). In general, our results proved at the genetic and protein levels that the GelMA/PAMAM-MA hydrogel can better promote the chondrogenic differentiation of rASCs in vitro than can the GelMA hydrogel.

### In vivo cartilage repair assays

**Fig. 5:**
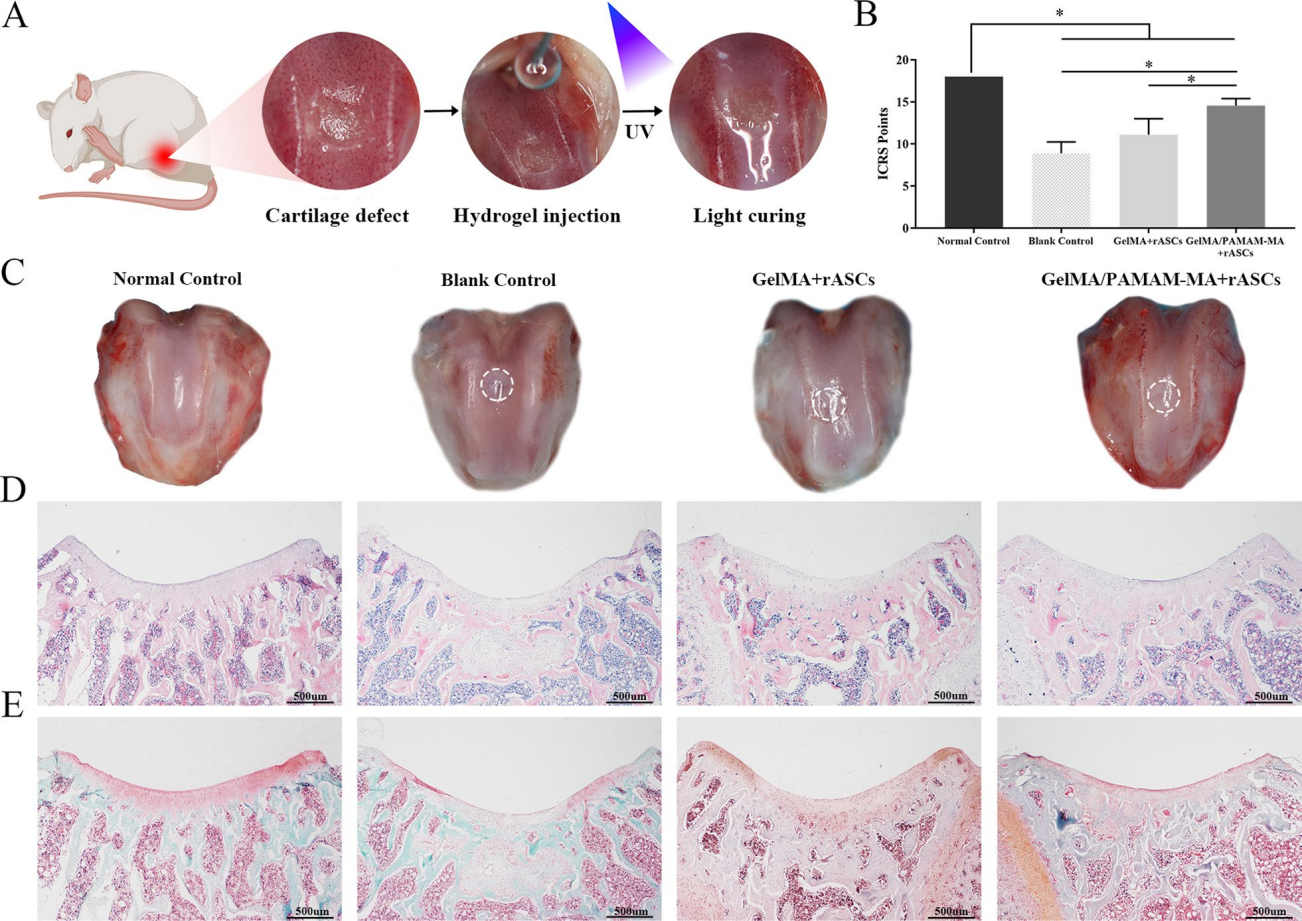
In vivo evaluation of cartilage repair in rats. **A** Schematic illustration of the surgical procedure in which rASCs were encapsulated in GelMA/PAMAM-MA hydrogel and injected into the articular cartilage defects of rats. **B** ICRS visual histological assessment score of the normal control group, blank control group, GelMA + rASCs group and GelMA/PAMAM-MA + rASCs group. **C** General views of rat knee joint specimens of the four groups obtained after repair for 8 weeks. **D** and **E** Histological images of H&E staining (**D**) and safranin-O/fast green staining (**E**) of rat cartilage defects in the four groups. Scale bars: 500 μm. Error bar: Mean ± SD, * represents *p* < 0.05

After comparatively analyzing the ability to induce cartilage differentiation of rASCs in vitro, we tested the ability of the two groups of hydrogels to promote cartilage regeneration in vivo. Hydrogels loaded with rASCs were injected into rat knee joint articular cartilage defects for 8 weeks of cartilage repair treatment (Fig. 5A). After they were loaded into the GelMA/PAMAM-MA hydrogel, GFP-labeled adenovirus-transfected rASCs were injected into cartilage defects. These rASCs could still be observed in the cartilage defect at 1 week, 2 weeks and 4 weeks after implantation (Additional file 1: Supplementary information, Fig. S2).

Gross morphological examining, histological staining and immunohistochemical staining were performed to assess the cartilage reparative capability of the two groups of hydrogels. Representative images in Fig. 5C show that the defects treated with rASCs-laden GelMA/PAMAM-MA hydrogels showed good neotissue filling with regular, surfaces and were indistinguishable from the surrounding tissues. The defects filled with rASCs-laden GelMA hydrogels displayed moderate new tissue coverage and surface regularity. The blank control group showed an irregular articular surface in the defect area, indicating insufficient filling and inadequate repair of the cartilage tissue.

H&E staining was performed to examine cartilage regeneration in the defect areas (Fig. 5D). When comparing the two groups of hydrogels, we found that the GelMA/PAMAM-MA hydrogel yielded better regeneration than the GelMA group since the defect almost disappeared, while a clear boundary between the neotissue and the adjacent natural cartilage could be observed in the GelMA hydrogel group. For the blank control group, the defect area was filled with fibrous tissues and a concave surface below the height of normal tissue could be observed, which confirms that cartilage was difficult to self-heal. Furthermore, safranin-O/fast green staining was used to assess the quality of the repaired cartilage (Fig. 5E). Representative images show that the GAGs content was deposited in the neotissue in the GelMA/PAMAM-MA + rASCs group, while no typical GAGs content was found in the defect areas of the other groups. The results of the ICRS visual histological assessment score showed that the GelMA/PAMAM-MA hydrogel group displayed significantly higher scores than did both the GelMA hydrogel group and blank control group (Fig. 5B).

**Fig. 6:**
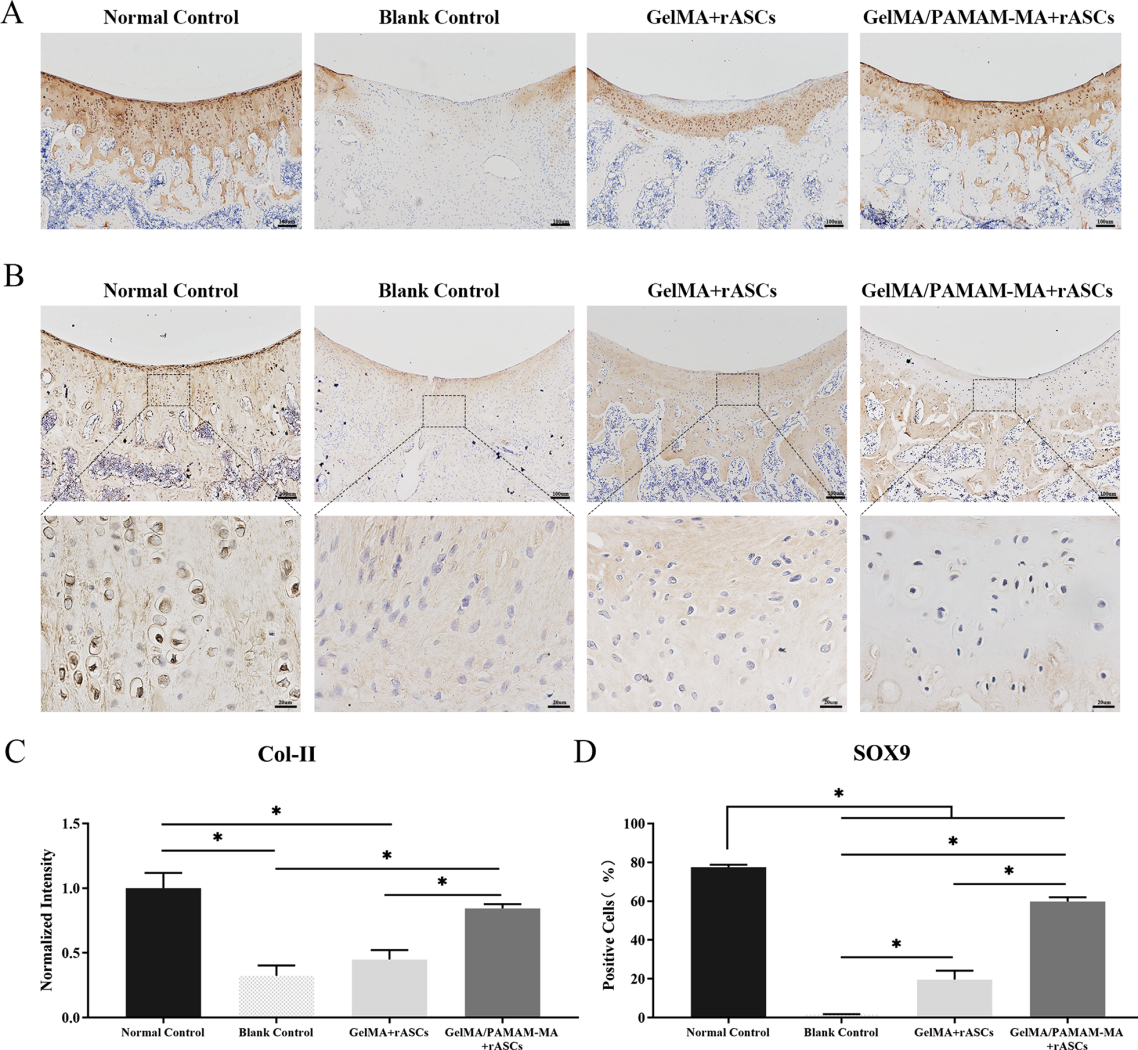
**A** Immunohistochemical staining of Col-II in the four experimental groups. Scare bars: 100 μm. **B** Immunohistochemical staining of SOX9 in the four experimental groups. Scare bars: above, 100 μm; below, 20 μm. **C** Quantification of Col-II expression in the four experimental groups. **D** Quantification of SOX9 expression in the four experimental groups. Error bar: Mean ± SD, * represents *p* < 0.05

In addition, we carried out IHC staining of cartilage-associated protein type II collagen and SOX9. The results of Col-II staining showed that more positively stained areas could be detected in the cartilage defect repair area of the GelMA/PAMAM-MA + rASCs group than in the GelMA + rASCs group, and the cartilage defect area of the blank control group was essentially the result of negative staining (Fig. 6A, C). Similarly, the percentage of SOX9-positive cells in the GelMA/PAMAM-MA + rASCs group was significantly higher than that in both the GelMA + rASCs group and the blank control group (Fig. 6B, D). Taken together, the above results indicated that, compared with the other treatments, the GelMA/PAMAM-MA hydrogel presented better in vivo cartilage repair ability.

## Discussion

In this study, we developed an injectable photocrosslinked hydrogel for ASCs delivery by combining GelMA with PAMAM-MA. The stability and mechanical properties of the photocrosslinked hydrogel were reinforced by incorporating PAMAM-MA into the GelMA hydrogel without damaging the biocompatibility. Moreover, the efficiency of the in vitro chondrogenic induction of the differentiation ability of the GelMA/PAMAM-MA hydrogel significantly improved compared with that of the GelMA hydrogel group. In addition, we proved that, compared with the other hydrogels, the GelMA/PAMAM-MA hydrogel has better efficiency in improving the in vivo cartilage regeneration.

Owing to their abundance, ready accessibility, nonimmunogenic and anti-inflammatory properties, ASCs are considered ideal sources of stem cells [38]. Some researchers have developed the preliminary clinical trials of the intra-articular injection of ASCs into osteoarthritis patients for cartilage regeneration therapy [39, 40]. However, there were some deficiencies in stem cell injection therapy, such as the adverse effects caused by easy diffusion and the inaccurate delivery of stem cells [12, 41]. Therefore, to accurately implant cells into the targeted cartilage defect areas and effectively induce the chondrogenic differentiation of ASCs, it is necessary to develop the cell delivery scaffolds with good biocompatibility, feasibility and chondrogenic induction ability [42]. In this regard, we have developed an injectable photocrosslinked hydrogel for ASCs delivery. This GelMA/PAMAM-MA hydrogel has been improved to have exert biocompatibility and cell affinity. Moreover, the incorporation of PAMAM-MA with GelMA not only improved the stability and mechanical properties of the hydrogel but also improved the chondrogenic differentiation ability of the encapsulated rASCs, which was verified by a series of physiochemical characterization tests as well as by in vitro and in vivo chondrogenesis studies.

The physicochemical properties of stem cell-loaded hydrogels are crucial in regulating cell viability and cell fate [43, 44]. The photocrosslinked GelMA hydrogel has good biocompatibility, cell affinity and plasticity, while its poor mechanical properties hinder its cartilage differentiation-inducing abilities. Therefore, we incorporated PAMAM-MA into the single component GelMA hydrogel to optimize the physiochemical abilities. By detecting the internal structure of hydrogels under two conditions of dry and wet conditions, we found that the addition of PAMAM-MA can reduce the pore sizes of the hydrogel and make the structure more compact and thoroughly distributed. In addition, the stability of the hydrogel was also improved, as the swelling ratio and the in vitro degradation rate of the GelMA/PAMAM-MA hydrogel greatly decreased compared with those of the GelMA hydrogel. These improvements enabled hydrogels to continuously immobilize stem cells to targeted locations, which is essential for effective cartilage defect repair. Moreover, the mechanical properties of hydrogels have been proven to play important roles in regulating stem cell fate, especially cell differentiation [45]. Previous studies have shown that stem cells tend to differentiate into adipose cells in a softer matrix, while in a stiffer matrix, they are preferred for chondrogenic and osteogenic differentiation [46]. Directional stem cell differentiation induction could be achieved by adjusting hydrogels to simulate the stiffness of the ECM of targeted cells [47]. Some researchers have modulated the stiffness of the photocrosslinked hydrogels by altering the degree of methacrylation and stiffer hydrogels can better induce the chondrogenesis of human ASCs [48]. However, the increase in the degree of methacrylation would damage cell vitalities. In this research, the mechanical properties of this composite hydrogel increased significantly with the addition of PAMAM-MA. The compressive modulus of hydrogels increased from 2.56 ± 0.26 kPa to 8.06 ± 1.39 kPa. Moreover, the cell viability of the encapsulated rASCs was not hindered.

After encapsulating rASCs into two groups of GelMA/PAMAM-MA hydrogels for 3D culture, we detected the biocompatibility of the hydrogels. The live/dead cell staining results showed that cells cultured in the two hydrogels had good vitality at all time points, and the addition of PAMAM-MA had a negligible effect on the viability of rASCs. In addition, we injected the rASCs-loaded GelMA/PAMAM-MA hydrogel into rat articular cartilage defects. These rASCs were transfected with GFP-labeled adenovirus. At 1 week, 2 weeks and 4 weeks after implantation, these rASCs could still be observed in the cartilage defect areas. Overall, the good biocompatibility of the photocrosslinked hydrogel was not affected by the addition of PAMAM-MA. Moreover, we analyzed the cell morphology of the encapsulated rASCs in the two groups of hydrogels. Cells loaded in the GelMA/PAMAM-MA hydrogel presented reduced spreading areas and a more spherical shape, similar to that of chondrocytes, which might be beneficial to chondrogenic differentiation of rASCs [49].

The physiochemical properties of hydrogels have been proven to play important roles in regulating stem cell fate, especially cell differentiation [19]. In view of this, we encapsulated rASCs into two groups of GelMA/PAMAM-MA hydrogels to determine their in vitro chondrogenic differentiation induction abilities. The expression of chondrogenic-related genes in rASCs was detected after 7 days of 3D culture. The expression levels of SOX9, ACAN and Col-II significantly increased in the GelMA/PAMAM-MA hydrogel, which indicated that the changes in physical properties of the GelMA/PAMAM-MA hydrogel induced by the addition of PAMAM-MA could directly affect the early chondrogenic differentiation abilities of rASCs. The expression of the hypertrophic chondrocyte-related gene Col-X showed no significant difference between the two groups, which was frequently mentioned as the main disadvantage of utilizing stem cells for cartilage repair [4]. Furthermore, the spatial distribution of GAGs and the protein expression level of SOX9 in the two groups of hydrogels further demonstrated that rASCs loaded in the GelMA/PAMAM-MA hydrogel had a better effect on chondrogenic differentiation in vitro. In general, compared with the GelMA hydrogel, the GelMA/PAMAM-MA hydrogel was proven to have better chondrogenic differentiation-inducing ability, which can be attributed to the optimization of the physiochemical and mechanical properties of the GelMA/PAMAM-MA hydrogel. However, we did not discuss in depth how the physical properties of hydrogels regulate the differentiation of stem cells, which was also a deficiency of this study. In our follow-up study, we can further conduct research on the regulation of stem cell differentiation by physical and mechanical signal transduction and provide a more reliable basis for the optimal design of stem cell delivery scaffold materials.

After the rASCs-loaded hydrogels were injected into rat articular cartilage defects for 8 weeks, we tested the repair effects by gross morphological examining, histological staining and IHC staining. Newly formed cartilage tissue filling and negligible boundaries were observed in the GelMA/PAMAM-MA hydrogel-filled cartilage defect areas, while the GelMA hydrogel group and blank control group showed irregular surfaces and clear boundaries between the neotissue and the adjacent natural cartilage. According to the following histological staining results of H&E staining, safranin-O/fast green staining and IHC staining (Col-II and SOX9), neocartilage tissue was formed in the GelMA/PAMAM-MA group; this tissue was tightly integrated with surrounding cartilage tissues. A mixture of cartilage and fibrous tissue was produced in the GelMA group and only fibrous-like tissue formed in the blank control group. In summary, the GelMA/PAMAM-MA hydrogel loaded with rASCs had a better cartilage repair effect than the GelMA hydrogel, which was consistent with the effects of our in vitro chondrogenic induction differentiation analysis.

## Conclusions

In this study, by combining GelMA and PAMAM-MA, we constructed an injectable photocrosslinked hydrogel to deliver rASCs for cartilage defect repair. The addition of PAMAM-MA significantly improved the uniformity of the internal network and the stability and mechanical properties of the GelMA hydrogel. Moreover, the biocompatibility of GelMA/PAMAM-MA hydrogels was not hindered. The capacity of the hybrid hydrogels to induce chondrogenic differentiation of rASCs in vitro was also significantly enhanced with the optimization of the physiochemical properties of the hydrogels. Finally, we verified the excellent ability of GelMA/PAMAM-MA hydrogels to promote cartilage regeneration through in vivo cartilage defect repair experiments. This injectable hybrid hydrogel is promising for application as a stem cell delivery tissue engineering scaffold and for eventually implementing a successful stem cell-based tissue engineering cartilage regeneration strategy.

## Supplementary Information

**Additional file 1**. Supplementary information, Table S1. Supplementary information, Fig. S1 and Supplementary information, Fig. S2.

## Data Availability

The datasets used and analyzed during the current study are available from the corresponding author on reasonable request.

## Abbreviations

GelMA: Gelatin methacrylate
MA: Methacrylic anhydride
PAMAM-MA: Methacrylic anhydride-modified poly(amidoamine)
PAMAM: Poly(amidoamine)
ASCs: Adipose-derived stromal/stem cells
3D: Three-dimensional
PEG-LA-DA: Poly(lactic acid)-b-poly(ethylene glycol)-b-poly(lactic acid) with acrylate end-groups
PBS: Phosphate-buffered saline
FTIR: Fourier transform infrared spectroscopy
Irgacure 2959: 2-Hydroxy-1(4-(hydroxyethoxy)pheny)-2-methyl-1-propanone
UV: Ultraviolet
SEM: Scanning electron microscopy
OCT: Optimal cutting temperature compound
Wi: Initial weight
Wd: Dry weight
rASCs: Rat adipose-derived stromal/stem cells
DMEM: Dulbecco’s modified Eagle’s medium
FCM: Flow cytometry
PDMS: Polydimethylsiloxane
DAPI: 4′,6-Diamidino-2-phenylindole
PFA: Paraformaldehyde
qPCR: Quantitative real-time polymerase chain reaction
SOX9: Sex determining region Y-box transcription factor 9
ACAN: Aggrecan
Col-II: Type II collagen
Col-X: Type X collagen
GAPDH: Glyceraldehyde-3-phosphate dehydrogenase
cDNA: Complementary DNA
ECM: Extracellular matrix
IF: Immunofluorescence
GFP: Green fluorescent protein
EDTA: Ethylenediaminetetraacetic acid
H&E: Hematoxylin and eosin
ICRS: International cartilage repair society
IHC: Immunohistochemical
DAB: 3,3′-diaminobenzidine
SD: Standard deviation
