# Supplementary material for: Dendrimer-modified gelatin methacrylate hydrogels carrying adipose-derived stromal/stem cells promote cartilage regeneration
Source: Stem Cell Res Ther. 2022 Jan 24;13:26. doi: 10.1186/s13287-022-02705-6 (PMC8785478; doi:10.1186/s13287-022-02705-6)
Supplement: Supplementary file 1 — Additional file 1. Supplementary information, Table S1. Supplementary information, Fig. S1 and Supplementary information, Fig. S2. [file 13287_2022_2705_MOESM1_ESM.docx]

**Stem Cell Research & Therapy**

**Supplementary information**

**Dendrimer-modified gelatin methacrylate hydrogels carrying adipose-derived stromal/stem cells promote cartilage regeneration**

Fengyi Liu^# 1,2,3^, Xu Wang^# 1,2,3^, Yuzhou Li^1,2,3^, Mingxing Ren^1,2,3^, Ping He^1,2,3^,Lu Wang^1,2,3^, Jie Xu^* 1,2,3^, Sheng Yang^* 1,2,3^, Ping Ji^* 1,2,3^.

1. College of Stomatology, Chongqing Medical University, Chongqing, China.
2. Chongqing Key Laboratory of Oral Diseases and Biomedical Sciences, Chongqing, China.
3. Chongqing Municipal Key Laboratory of Oral Biomedical Engineering of Higher Education, Chongqing, China.

**Corresponding authors:**

Prof. Ping Ji, E-mail: jiping@hospital.cqmu.edu.cn.

Prof. Sheng Yang, E-mail: 500283@cqmu.edu.cn.

Dr. Jie Xu, Email: xujie@hospital.cqmu.edu.cn.

# Fengyi Liu and Xu Wang contributed equally to this research.

Figure S1. Frozen section images (A) and statistical analysis (B) of the GelMA and GelMA/PAMAM-MA hydrogel’s pore sizes in wet condition. Scare bars: 200 μm. Error bar: Mean +/- SD, * represents *p* < 0.05.


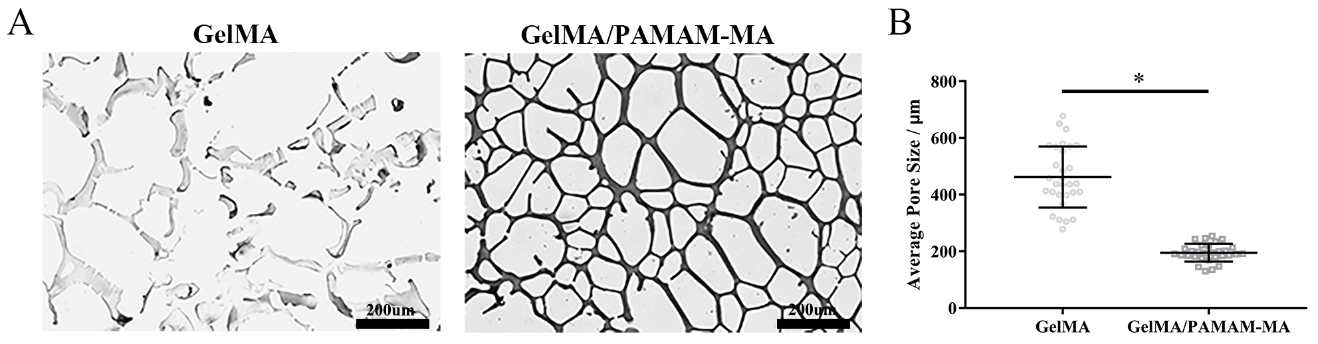


Figure S2. *In vivo* tracing of GFP-adenovirus transfected rASCs for rat knee joint cartilage defects repair at 1, 2 and 4 weeks. Green fluorescence indicated GFP-adenovirus transfected rASCs and blue fluorescence indicates cell nucleus. Scare bars: 75 μm.


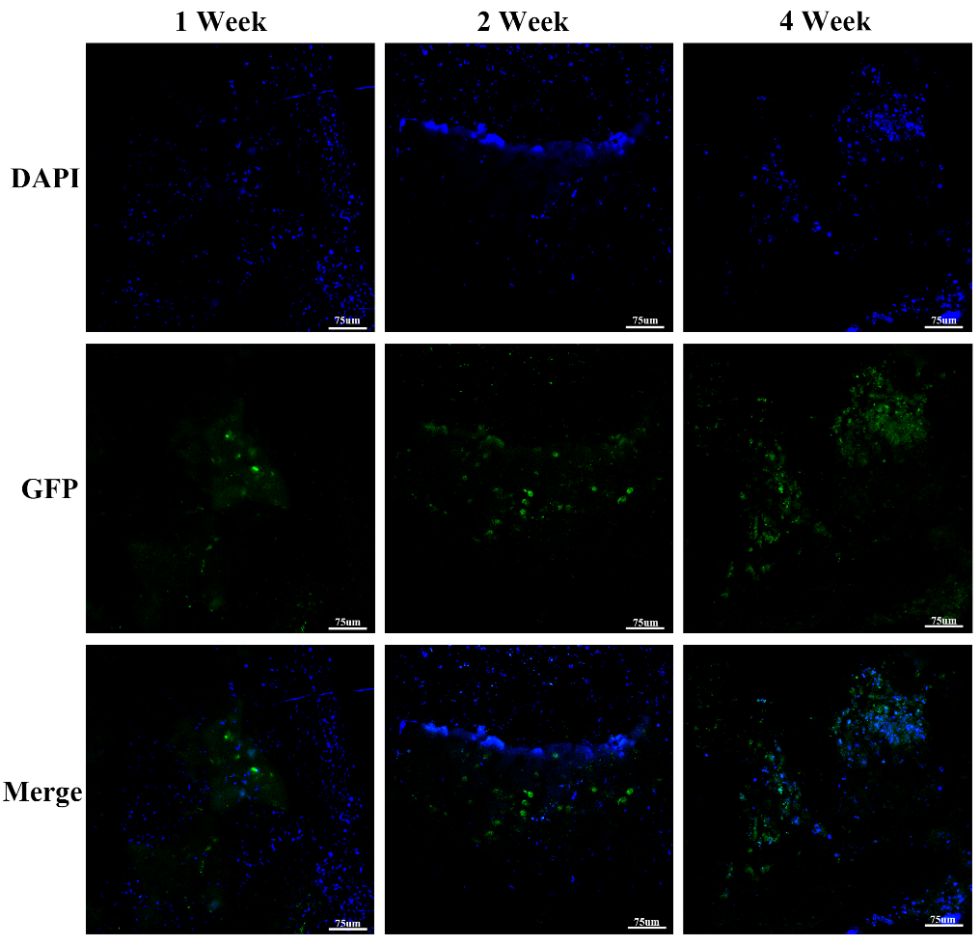


Table S1. Primer sequences of housekeeping gene and chondrogenesis related genes.

| Gene | Sense primer | Anti-sense primer |
| --- | --- | --- |
| GAPDH | AAGTTCAACGGCACAGTCAAGG | CGCCAGTAGACTCCACGACATA |
| SOX9 | CGCACATCAAGACGGAGCAA | GGTTGTAGTGCGGAAGGTTGAA |
| ACAN | ACCTCTGCCTCCCGTGAAAC | GTGCCTGCATCTATGTCGGAG |
| Col-II | GCTCCCAGAACATCACCTACCAC | GCCATCCTTCAGGGCAGTGTA |
| Col-X | GGATGCCTCTTGTCAGTGCTAAC | TGGGTCATAGTGCTGCTGCC |
